# Dynamics of study strategies and teacher regulation in virtual patient learning activities: a cross sectional survey

**DOI:** 10.1186/s12909-016-0644-y

**Published:** 2016-04-23

**Authors:** Samuel Edelbring, Rolf Wahlström

**Affiliations:** Department of Medical and Health Sciences, Division of Community Medicine, unit of Medical Education, Linköping University, SE-581 83 Linköping, Sweden; Department of Learning, Informatics, Management and Ethics, Karolinska Institutet, Stockholm, Sweden

**Keywords:** Study strategies, Self-regulation, Teacher guidance, Virtual patients

## Abstract

**Background:**

Students’ self-regulated learning becomes essential with increased use of exploratory web-based activities such as virtual patients (VPs). The purpose was to investigate the interplay between students’ self-regulated learning strategies and perceived benefit in VP learning activities.

**Method:**

A cross-sectional study (*n* = 150) comparing students’ study strategies and perceived benefit of a virtual patient learning activity in a clinical clerkship preparatory course. Teacher regulation varied among three settings and was classified from shared to strong. These settings were compared regarding their respective relations between regulation strategies and perceived benefit of the virtual patient activity.

**Results:**

Self-regulation learning strategy was generally associated with perceived benefit of the VP activities (*rho* 0.27, *p* < 0.001), but was not true in all settings. The association was higher in the two strongly regulated settings. The external regulation strategy did generally associate weakly with perceived benefit (*rho* 0.17, *p* < 0.05) with large variations between settings.

**Conclusions:**

The flexible student-autonomous appeal of virtual patients should not lead to the dismissal of guidance and related course activities. External teacher and peer regulation seem to be productive for increasing learners’ perceived benefit. Awareness of the interplay among teacher regulation (external) and various study strategies can increase the value of flexible web-based learning resources to students.

**Electronic supplementary material:**

The online version of this article (doi:10.1186/s12909-016-0644-y) contains supplementary material, which is available to authorized users.

## Background

Digital web-based learning materials show greater potential for autonomous student use than other types of course materials. Students decide on the pace and sequence of interaction, and access course content following their perceived needs [1]. Time and place of learning are not determined, often resulting in students using these materials “off hours” and “off campus” [2, 3]. However, even if students control the time, place, and manner in which these materials are used, teachers and course designers usually set directions or requirements for their use. Thus, a relationship is created between student autonomy, flexibility of learning resources, and teacher guidance. An increased awareness and understanding of this relationship is needed to support students’ learning [4, 5].

Research on students’ study strategies has often centred on the concept of self-regulated learning. Self-regulated learning is related to motivation, autonomy, and control of students’ own learning processes [6–9]. Medical education scholars have recently began to use this concept to raise awareness about the complex dynamics of guidance in the learning process [6, 10, 11]. Self-regulation has been used to describe students’, and teachers’, preferences on a continuum, with a highly didactic and regulated situation at the one end, and a highly autonomous situation at the other end, where students make their own decisions about how to study [12]. The ability to self-regulate is crucial for learning using the “anytime anyplace” online learning environments that are increasingly used in education [13–15]. Self-regulation in learning is regarded as both a necessary attribute for academic success and as a quality that can, and should, be developed by students in order to reach future independence in lifelong learning [10, 16]. Researchers have emphasized the roles of guidance, feedback, and support provided by teachers in the development of self-regulation [9, 17]. Consequently, both students and educators need to consider the manner in which self-regulation is supported in their contexts and how it is matched by external regulation.

In the formal learning environment, external regulation is exerted via course requirements, and directions and guidance from teachers [18]. Although they appear to be conceptually different and are even seen as extremes on a continuum, self-regulation and external regulation are not mutually exclusive, and they can be expressed dynamically in the learning situation [12, 16, 18, 19]. This dynamic has been described by Vermunt and Verloop (1999) as a model in which students’ degrees of self-regulation are matched with teachers’ degrees of external regulation (Table 1). The interplay between teachers’ regulation and students’ self-regulation can influence both forms of regulation, either constructively or destructively, depending on teaching and studying preferences [18]. The ideal learning climate is one in which students’ self-regulation preferences are matched by teachers’ degrees of regulation or when the combination creates the kind of friction that is conducive to learning [18]. In a Vygotskian sense this matching provides a scaffold for the student, which is then gradually dismantled as the learner construct his or her own stable knowledge and proficiency [20]. A transition is thus sought over time from strong to shared teacher regulation in harmony with students’ development of knowledge and self-regulation [10].

**Table 1:** Interplay between levels of teacher regulation and levels of student regulation in learning processes; adapted from Vermunt & Verloop (1999)

*Degree of student regulation of learning*	*Degree of teacher regulation of learning*		
	*Strong*	*Shared*	*Loose*
*High*	Destructive friction	Destructive friction	Congruence
*Intermediate*	Destructive friction	Congruence	Constructive friction
*Low*	Congruence	Constructive friction	Destructive friction

Teachers generally aim to design and align study activities with the intended learning outcomes in order to optimize student learning [21]. According to the principle of student-centredness, learning occurs as a consequence of learners’ activities rather than resulting directly from teachers’ words and actions. Learners are viewed as active processors and creators of knowledge rather than passive recipients of facts. Emphasis on teacher-directed learning (strong external regulation) might lead to inert knowledge, in that students might learn readymade abstract theory without processing or applying the knowledge to any great extent [22]. An illustrative example is the case in which medical students have difficulties applying biomedical knowledge when they first encounter clinical practice [7, 23].

From the viewpoint of flexible learner-directed situations for applying clinical knowledge, computerized virtual patients (VPs) constitute an interesting learning resource [24]. VPs in terms of interactive patient scenarios [25] allow students to perform virtual investigations, gather clinical information, and decide on diagnoses and managements, thereby learning diagnostic skills and enhancing their biomedical knowledge in a virtual clinical context. The VP resource allows for autonomous student use but can be implemented with various degrees of teacher regulation [2, 24, 26]. Empirical evidence on optimal relation between teacher direction and VP benefit is lacking. However, according to studies on use of clinical simulation in similar contexts, teacher guidance is influential for students’ learning progress [27].

The aim of this study was to enhance our understanding of the relationships between students’ self-regulated learning strategies and teacher regulation in the context of computerised VP activities. It was anticipated that regulation strategies, foremost the self-regulated learning strategy, influence the perceived benefit of VP activities. More specifically, two research questions were formulated: In which ways are students’ self-regulated learning strategies (a) related to the perceived benefits of VP learning activities, and (b) to teacher regulation of such learning activities?

## Methods

### Empirical context

The empirical data for this analysis were gathered in a cross-sectional survey on VP learning activities in the setting of a clerkship preparatory course during spring 2009. A previously reported part of this study focused on the use of follow-up activities in relation to the students’ perceived benefit of VPs and their use in the aforementioned setting [28]. The present analysis adds data on students’ study strategies, and the relationship between these and the perceived benefit. Because of variations in degrees of teacher regulation of the VP activities between the settings, the correlations were also compared with regards to levels of external regulation.

Students worked in a self-directed manner with four VP cases presenting symptoms that were aligned with course topics and intended learning outcomes. The Web-SP virtual patient platform was used for delivering the VP cases online [29]. Time was allocated for self-study of VPs during the course, but a specific time or place was not assigned for this purpose. In general, students spent about 50 min (based on log-files) on each accessed case to record patient history, perform physical examination, and present diagnosis and differential diagnoses.

Four settings of the same course were analysed. The VP activity formed an integral part of the courses, and thus mandatory in all settings, but the external regulation of the VP activity differed between the settings. This variation was naturally occurring, i.e. not by the researchers’ design. The authors synthesized the main characteristics of the pedagogical framework surrounding the VP learning activity into degrees of teacher regulation (Table 2). Setting 1 was characterized by *shared teacher regulation* [18] of the web-based case activity, but it required students to attend a seminar in a lecture hall, where a clinician presented the case on a large screen and asked questions pertaining to the case. Setting 2 exerted *stronger teacher regulation* by requiring students to participate in four group seminars, where each student was expected to participate actively. Setting 3, too, was *strongly externally (teacher and peers) regulated*, notably because of the requirement that students should present their cases to each other group-wise, facilitated by two clinicians. One setting (Setting 0) was found to be regulated loosely because of the lack of requirements related to the VP activity, other than the activity being mandatory. This setting was discarded from the analysis owing to low participation in the VP activity (49 %) and low (19 %) response rate to questionnaires, which make statistical association analysis less meaningful.

**Table 2:** Characteristics of seminars and teacher regulation for three analysed course settings

	*Settings (No. of participants using virtual patients/tot. course participants)*		
*External regulation aspects*	1 *n = 31/48*	2 *n = 69/72*	3 *n = 62/70*
Group size in virtual patient (VP) seminars	≈48	≈12	≈12
Number of VP seminars	1	4	1
Length of VP seminar	1.5 h	1 h each	1.5 h
No. of clinicians present per session	3	1	2
Number of VP cases discussed	2	4	2
Required from the students	Know the cases	Know the cases	Be able to present cases
Overall level of teacher regulation of learning	Shared	Strong	Strong

### Measures

A questionnaire addressing regulation strategies related to and perceptions on VP activities were distributed in the latter part of the course, but before the examination. Students’ regulation strategies were analysed using the regulation strategy section of Vermunt’s Inventory of Learning Styles [30]. These scales have been used widely and documented in research on students’ learning patterns [31, 32]. There were three scales of *self-regulation, external regulation*, and *lack of regulation* comprising 28 items in total. The psychometric properties of the scales in this setting show adequate internal consistency, with Cronbach’s alpha (α) ranging from 0.65 to 0.82 [33].

Other questionnaire items were formulated by the first author in collaboration with other VP and education experts in order to cover aspects of how VPs were being used, for example, estimated time of VP case work, location (at home or in campus), and statements, primarily, on the perceived benefit of VP-assisted learning activities. Twenty items were graded from “not at all” to “to the greatest extent”, represented by numbers 1–5. By grouping the items using Mokken exploratory scale analysis [34], three outcome variables were formed (four items were discarded for not reaching the Mokken criteria of a homogeneity coefficient >0.3). The three variables were labelled *perceived benefit of virtual patient use* (11 items, α = 0.89), *wish for more guidance* (3 items, α = 0.73), and *wish for more assessment and feedback* (2 items, α = 0.74) (see Additional file 1). The primary outcome variable “*perceived benefit of virtual patient use*” was used to consider various aspects of the perceived benefit of the VP activities from the students’ perspective. The “*wish for more guidance*” variable was concerned with the need for enhanced structural guidance before the VP activity. The “*wish for more assessment and feedback*” variable comprised items regarding assessment and feedback after the VP activity. The questionnaire was piloted before the study in two of the four settings, and its wording was refined slightly after interviews with students.

### Analyses

Associations between learning strategy scales (*self-regulation, external regulation*, and *lack of regulation*) and VP variables (*perceived benefit of virtual patient use*, *wish for more guidance* and *wish for more assessment and feedback)* were estimated by Spearman’s *rho*. To analyse several variables in combination, a multiple regression model was used. Assumptions of linearity, equal variance, and normality were investigated via analysis of residual plots and quartiles of the regression model, and were found to be adequate. Differences on scale scores in terms of category variables and course settings were analysed using ANOVA. Post hoc tests were performed to determine settings that differed from each other using *Tukey’s Honest Significant Difference* test with adjusted *p*-values. All analyses were performed using the statistical package *R*, version 2.13.0. A significance level of 0.05 was chosen.

## Results

The analysis displayed a general association between the self-regulation learning strategy and the perceived benefit of virtual patient use. However, the associations between regulation strategies and perceived benefits varied across settings.

### Regulation strategies and perceived benefits

**Fig. 1:**
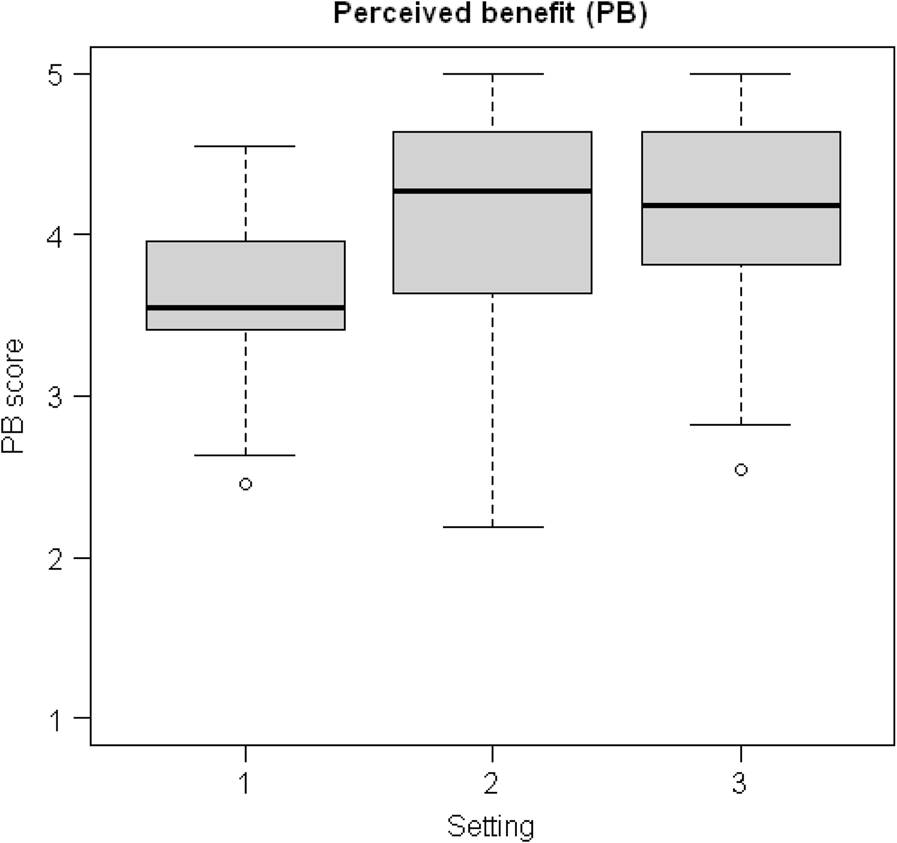
Box plot of *perceived benefit of virtual patients* in the three settings

The questionnaire was completed by 150 (79 %) out of 190 eligible participants (discarding Setting 0). The student groups in the three settings displayed equal levels of regulation strategy scores (Table 3). The *perceived benefit of VP use* was higher in both the setting with strong teacher regulation and the setting with strong teacher and peer regulation (settings 2 and 3, respectively), than in the setting with shared regulation (setting 1) (Fig. 1). In all groups combined, there was a moderate association between *self-regulation* and *perceived benefit of VP use (rho* 0.27, *p* < 0.001). However, this association varied from none in setting 3 to *rho* 0.38 (*p* < 0.05) in setting 1 (Table 4). In all groups combined, there was a weak positive association between *external regulation* and *perceived benefit of VP use* (*rho* 0.17, *p* < 0.05). This association was negative in setting 1 (*rho −*0.39, *p* < 0.05) and positive in the two other settings (*rho* 0.32, 0.36, respectively, in settings 2 and 3; both *p* < 0.05). The association between *wish for more guidance* and *perceived benefit of virtual patient use* was negative in all settings, whereas the association between *wish for more guidance* and *lack of regulation* was positive throughout the sample (Table 4).

**Table 3:** Mean (standard deviation) of summed scores for study strategy variables, perceived benefit and wish for guidance and feedback regarding the virtual patient learning activity

	Setting 1	Setting 2	Setting 3	Combined	ANOVA difference*
	*n* = 27	*n* = 65	*n* = 58	*n* = 150	
Self-regulation strategy	2.58 (0.76)	2.90 (0.79)	2.81 (0.71)	2.81 (0.76)	0.18
External regulation strategy	3.27 (0.54)	3.14 (0.49)	3.08 (0.57)	3.14 (0.53)	0.31
Lack of regulation strategy	2.59 (0.51)	2.5 (0.61)	2.38 (0.63)	2.47 (0.69)	0.28
Perceived benefit of VP use	3.6 (0.51)	4.12 (0.63)	4.18 (0.53)	4.05 (0.61)	**<0.001**
Wish for more guidance	2.42 (0.74)	3.40 (0.96)	2.00 (0.65)	2.68 (1.04)	**<0.001**
Wish for more assessment and feedback	2.00 (0.93)	2.22 (1.09)	2.06 (1.08)	2.12 (1.06)	0.57

*p-values. Significant values in bold

**Table 4:** Associations between regulation strategy scales and *Perceived benefit of VPs*, *Wish for more guidance* and *Wish for more assessment and feedback*

	Settings			
Paired variables	1 (*n* = 27)	2 (*n* = 65)	3 (*n* = 58)	All (*n* = 150)
Perceived benefit – Self-regulation strategy	0.38*	0.29*	0.04	0.27***
Perceived benefit – External regulation strategy	−0.39*	0.32**	0.36**	0.17*
Perceived benefit – Lack of regulation strategy	−0.41*	0.08	0.10	−0.09
Wish for more guidance – Perceived benefit	−0.31	−0.44***	−0.26*	−0.26***
Wish for more guidance – Self-regulation strategy	0.07	−0.07	0.13	0.09
Wish for more guidance – External regulation strategy	0.14	−0.10	−0.22	0.05
Wish for more guidance – Lack of regulation strategy	0.34	0.23	0.36	0.27***
Wish for more assessment and feedback –Perceived benefit	0.09	0.05	0.34**	0.13
Wish for more assessment and feedback – Self-regulation strategy	0.38	0.06	0.02	0.09
Wish for more assessment and feedback – External regulation strategy	0.007	−0.01	0.07	0.03
Wish for more assessment and feedback – Lack of regulation strategy	0.33	0.008	−0.06	0.02

Associations measured by Spearman’s Rho. Significant values in bold. ***p < 0.001, **p < 0.01, *p < 0.05

### Regulation strategies and need for teacher regulation

Study time per virtual patient case was found to be correlated to *external regulation* in the overall sample (*rho* 0.24, *p* = 0.004). This relation was true for the two highly regulated settings (*rho* 0.33, 0.29 for settings 2 and 3, respectively, both *p* < 0.05) but not for the shared regulated setting 1 (*rho* 0, n.s.). Neither *self-regulation* nor *lack of regulation* were found to be correlated to the study time per virtual patient case. No differences were found in association between regulation strategy scores and location of use (at home or on campus), nor choice of studying using VPs individually or collaboratively. Among all single items in the VP part of the questionnaire, two items scored maximum (median 5): a) “Does it train your ability to reach diagnoses?” and b) “I appreciate the flexibility of working when and where it suits me.”

Combined analyses of independent variables (*settings*, *self-regulation, external regulation, wish for more guidance,* and *wish for more assessment and feedback)* using a regression model showed that they influenced *perceived benefit of virtual patient use* (R^2^: 0.36, *F*(6, 143) = 13.16, *p* < 0.001). The scores for *lack of regulation* were redundant in the model because of a non-significant influence (regression coefficient: 0.05, *p* = 0.52). Setting 2 was selected as the reference setting in the regression model because the regulation strategies’ relationships with *perceived benefit of virtual patient use* in this setting were most closely matched to the ones found when all settings were combined. A significant interaction effect was detected between *external regulation* and *settings* when these variables were allowed to interact in the model (interaction coefficients: *Setting 1*—0.67, *p* < 0.01; *Setting 3*—0.14, n.s.). Including this interaction in the model also led to an increase in the explained variance (R^2^) in the model (R^2^: 0.40, *F*(8, 141) = 16.42, *p* < 0.001).

## Discussion

The study findings contribute to more profound understanding of the interplay between teachers’ and students’ regulation of learning activities. The interplay is expressed foremost in associations between the perceived benefit of virtual patient use and the study strategies of self-regulated learning and external regulation of learning. Overall, *self-regulation* was expressed moderately together with *perceived benefit of virtual patient use*. A positive association was also expressed between *external regulation* and *perceived benefit of virtual patient use*. However, when analysing the settings separately considering varying degrees of teacher regulation, a pattern could be discerned. In the setting with the lowest teacher regulation (characterised as “shared”), self-regulation was expressed positively in relationship with perceived benefit of virtual patient use, and external regulation was expressed negatively (Table 4). In the setting characterized by the highest teacher regulation (“strong,” setting 3), this pattern was reversed, with the expression of high *external regulation* and no *self-regulation* in relation to *perceived benefit of virtual patient use*. According to the theory of dynamics between teachers’ and students’ regulation of learning, this dynamic can either be dominated by teachers or students or shared between students and teachers [10, 18]. Following this theory, students in highly regulated settings need undertake the initiative to engage fully in learning tasks. By contrast, in loosely regulated settings, they risk not engaging at all. Setting 0 (not analysed) in this study presents such an example in which most students did not engage in the virtual patient-assisted learning activity. The perceived benefits were markedly higher in the more strongly regulated settings 2 and 3 (Fig. 1). In addition to teacher-regulation elements, students in these settings seemed to have used a mix of *self-regulation* and *external regulation*, with the lowest degree of *self-regulation* in setting 3.

The main difference between the two more strongly regulated settings 2 and 3 was that students in setting 3 were required to present the cases to a peer student group, whereas the students in setting 2 were not expected to do so. The requirement of case presentation in this setting led students to process the cases to greater extents than required in the other settings, even though additional time was allocated for case discussion in teacher-led seminars conducted in setting 2. This extra processing required group coordination and was, consequently, regulated by peers within the seminar group. This regulation may be productive for meaning-orientation in learners’ study focus. In this highly regulated setting, *perceived benefit of virtual patient use* was associated with *external regulation* not with *self-regulation*.

Analysis of the regression model suggested that both self-regulation and teacher regulation were associated with students’ perception of the benefits of VP learning activities. The estimates of the influence of *external regulation strategy* and *setting* in the regression model were greater than the influence of *self-regulation* when considering the influence of all variables on *perceived benefit of VP use.* A plausible interpretation is that the external regulation strategy was indeed important for the perceived benefit of the virtual patient learning activities. The influences of external regulation and teaching strategy on the degree of e-learning use and learning outcomes have been identified previously [35]. The possible greater influence of the external regulation strategy is surprising given the prominent role of self-regulation in web-based activities, as stated in the literature [15]. The combined influence of self- and external regulation strategies highlights that empowerment of autonomous learners may co-exist with students’ responsiveness to teachers’ involvement and guidance in the learning activities.

Therefore, teacher regulation and individual study strategies should be considered when designing VP-assisted learning activities, as well as from the perspective of developing self-regulation in learners [16, 36]. Self-regulation should be viewed from a wide, integrated perspective to nurture future lifelong autonomous learners [6, 12]. As is routine for integration of new elements into a course, the success of VP activities is often demonstrated by eliminating other course activities and ensuring that teachers convey the importance and alignment of VP activities with the intended learning objectives [21, 37]. Teachers introducing web-based technology resources as part of such an integration strategy must face the apparent conflict presented by the flexible opportunities of freedom in time and space and the benefits of regulating learning activities with assignments, feedback, and follow-up. The students in this study appreciated the flexibility in terms of time and space for self-study, but the combination with teacher-regulated discussion seemed to provide added benefits for learning. These discussions may also have contributed an increased perception of relevance of the VP activity in relation to the course examination. In the course settings, external regulation was exerted in several dimensions, for example, course requirements and participation in teacher-led seminars. External regulation can also to pertain to the specific interface design of the VP system itself. That means what is possible to do within the system, expectations that it conveys to students and built-in guidance. The VP system used here (Web-SP) features little built-in regulation and provides many possibilities, e.g., virtual lab-tests and physical examinations in no specified order.

The real-world setting with natural variations in the external regulation of the same course provided an opportunity to study the relationship between external regulation and self-regulation in authentic settings with conditions difficult to otherwise arrange in an ethical way. There are, however, some limitations worth mentioning. We did not control the occurrence of variations in teaching, and neither was it possible for us to observe and record all differences that could influence the students’ perception of the VP activity and the course settings. Thus, the comparison between settings should be interpreted with caution because of other possible variations than the ones highlighted here. Furthermore, the regression analysis is weakened by the fact that data levels of *settings* were not fully compatible with the variables based on summed scales of ranked items.

Some implications for the design of VP-learning activities can be derived from these findings. Teachers should provide external regulation supportive of VP activities. The higher values of *perceived benefit of virtual patient use* in both settings 2 and 3 indicated that external regulation is important for students to perceive virtual patient use as beneficial for their learning. The external regulation in these settings comprised essentially follow-up seminars, which, in turn, can be designed to support various means of further engaging students in deep learning and set the virtual patient cases in a broader clinical context. Moreover, peer presentations seemed to be a productive part of the external regulation. Directing students to further engage in VP case processing seemed to enhance the benefit of the VP-assisted learning activities, although it decreased the flexibility afforded by the VP technology to some extent. Future research should investigate ways of using external regulation to support learning, while using the benefits of flexible learning resources such as VPs. The processing characteristics of VPs should be considered when optimizing assignments and teacher regulation to put an autonomous student in charge of the virtual clinical reasoning process.

Ideally, the external regulation should be individually matched to levels of knowledge, progression and degree of autonomy with each student. However, regulation in a personally adapted scaffolding sense [20] can not be universally provided for heterogeneous student groups using open ended and exploratory methods like VPs. To some extent the regulation during seminars and peer regulation seem to fill this function but the teachers’ facilitating skills and awareness to regulation needs are still at the centre of this process. Consequently, there is much to gain from developing facilitator skills in successful guidance of exploratory and experiential learning and provide access to skilled facilitators in relation to VP activities [38].

Our assumption that the self-regulated learning strategy would influence the perceived benefit of the virtual patient activities was somewhat supported on a general level. However, influences from teacher and peer regulations seemed equally important, if not more important. The influence of external regulation highlights the importance of considering not only individual regulation strategies but also directing focus towards teacher and peer regulations supporting student learning with virtual patients.

## Conclusions

Web-based resources such as virtual patients have a flexible student-autonomous appeal. The ability to benefit autonomously from these activities in learning should not be overestimated but should be matched with teacher guidance. The use of follow-up activities such as different forms of peer and teacher feedback seems to enhance the value of virtual patient learning activities. Consequently, teachers must face the conflict between the benefits of the flexibility of web-based technologies, namely, freedom in time and space, and the benefits of teacher regulation of learning activities via, for example, assignments, feedback, and follow-up.

### Ethics approval and consent to participate

The regional ethical board in Stockholm approved of the study (No: 2008/822-31/5). Each questionnaire contained information on the study and that the participation was anonymous and voluntary.

### Consent for publication

Not applicable.

### Availability of data and materials

Data and materials are available with the first author.

## Additional file

Additional file 1: Items included in variables *Perceived benefit of virtual patient use*, *Wish for more guidance*, and *Wish for assessment and feedback* (items translated from Swedish). (DOCX 17 kb)

## Abbreviations

n.s.: not significant
VP: virtual patient
