# Supplementary material for: Dynamics of study strategies and teacher regulation in virtual patient learning activities: a cross sectional survey
Source: BMC Med Educ. 2016 Apr 23;16:122. doi: 10.1186/s12909-016-0644-y (PMC4842289; doi:10.1186/s12909-016-0644-y)
Supplement: Additional file 1: — Items included in variables Perceived benefit of virtual patient use, Wish for more guidance, and Wish for assessment and feedback (items translated from Swedish). (DOCX 17 kb) [file 12909_2016_644_MOESM1_ESM.docx]

## Items included in variables *Perceived benefit of virtual patient use*, *Wish for more guidance*, and *Wish for assessment and feedback* (items translated from Swedish).

**Variable: *Perceived benefit of virtual patient use* (11 items, Cronbach’s alpha: 0.89)**

What did the virtual patient activity contribute?

Did it help you to perform better on the examination?

Is it beneficial for your future professional practice?

Did it influence your understanding of the topics in the course?

Did it facilitate the learning of new facts about symptoms and diagnoses?

Did it train your ability to reach diagnoses?

Did it connect the topics you study to reality?

Did it cause you to use a greater number of Internet sites related to the subject?

Do you prefer web cases to paper-based patient cases?

Please state to what degree you appreciate different aspects of web-case activity:

Getting acquainted with a greater number of patient cases

Leading an investigation independently

Getting an overview of diagnostic and investigation possibilities

**Variable: *Wish for more guidance* (3 items, Cronbach’s alpha: 0.73)**

Has it been clear to you what you can expect of the web cases? (reversed scoring)

Has it been clear to you how the web cases relate to the course? (reversed scoring)

Did you wish for increased guidance about working with the web cases?

**Variable: *Wish for more assessment and feedback* (2 items, Cronbach’s alpha: 0.74)**

Did you wish for a greater number of course assignments relating to the web cases?

Did you wish for more seminar discussions of the web cases?
